# Ultrafiltered Sweet Buttermilk as Additive Replacer in Ice Cream Production

**DOI:** 10.3390/foods13193134

**Published:** 2024-09-30

**Authors:** Mihaela Ivanova, Marcello Alinovi, Mariya Dushkova, Luca Trublet, Massimiliano Rinaldi, Davide Barbanti, Emma Chiavaro, Zhana Petkova, Olga Teneva, Nikolay Menkov

**Affiliations:** 1 Department of Milk and Dairy Products, University of Food Technologies, 26, Maritsa Blvd., 4002 Plovdiv, Bulgaria; mihaela_18bg@abv.bg; 2 Department of Food and Drug, University of Parma, Parco Area delle Scienze, Pad. 33, 43124 Parma, Italy; marcello.alinovi@unipr.it (M.A.); massimiliano.rinaldi@unipr.it (M.R.); davide.barbanti@unipr.it (D.B.); 3 Department of Process Engineering, University of Food Technologies, 26, Maritsa Blvd., 4002 Plovdiv, Bulgaria; m_dushkova@uft-plovdiv.bg (M.D.); nimenkov@uft-plovdiv.bg (N.M.); 4 Claude Bernard University Lyon 1, Institut Universitaire de Technologie Lyon 1, 155 rue Henri de Boissieu, 01000 Bourg en Bresse, France; luca.trublet@gmail.com; 5 Department of Chemical Technology, University of Plovdiv “Paisii Hilendarski”, 24 Tzar Asen Street, 4000 Plovdiv, Bulgaria; zhanapetkova@uni-plovdiv.net (Z.P.); olga@uni-plovdiv.bg (O.T.)

**Keywords:** ultrafiltration, phospholipids, melting, additive replacement, by-product

## Abstract

Sweet buttermilk, a by-product of butter production, remains highly underutilized despite containing some relevant components (i.e., phospholipids) that may have a high biological value and may exert some positive technological functions. The aim of this study was to investigate the possibility of using ultrafiltered (UF) sweet buttermilk at different volume reduction ratios (3 and 5) to replace emulsifiers and/or stabilizers in the production of a novel clean-label ice cream formulation made with sweet buttermilk-based mixtures. The functional, thermo-rheological, and sensory profile of four types of ice creams was investigated. Increasing the degree of sweet buttermilk concentration positively influenced the overrun values and at the same time improved the ice cream’s resistance to melting. Also, the thermo-rheological profile during melting was influenced by the presence of UF buttermilk. These differences in techno-functional properties were probably partly caused by the different total phospholipids content caused by UF buttermilk. Some sensory properties (i.e., structure, consistency) were positively related to the utilization of UF buttermilk, while aroma and taste were negatively influenced. This study demonstrated that UF buttermilk can be used as an additive replacer in ice cream production because it enhances the structural and rheological properties of the final product.

## 1. Introduction

Nowadays, the opportunities for the development of new products with increased health characteristics are increasingly sought. The modern consumer is oriented towards “clean-label” products [1]. In this view, more and more manufacturers are looking for ways to replace artificial additives with natural ones [2]. Milk and dairy products are an excellent source of several valuable nutrients—fats, proteins, carbohydrates, minerals, as well as some vitamins and other minor components. During the processing of milk into various dairy products, some of these components are separated into secondary dairy products or the so-called “by-products”. In the production of sweet butter from cream that undergoes only physical ripening without the addition of starter cultures and biological ripening, sweet buttermilk is separated. The processing of buttermilk by-products is a current direction in the field of sustainable technologies, since according to Directive 2008/98/EC [3], its processing and re-utilization is mandatory. It is well known that in this by-product, a significant amount of phospholipids and other minor components (lactose, minerals, and proteins) are present [4]. These compounds are bioactive and characterized by high biological value. Phospholipids are important compounds in the cell membranes that play an essential role in organisms’ biological processes. The most common components among the phospholipids are phosphatidylcholine and phosphatidylethanolamine, and sphingomyelin is the main one among the sphingolipids. It is established that the intake of a certain amount of these compounds with the food may have a health-promoting effect. They are associated with benefits for cardiovascular health through reducing cholesterol levels and improving lipid profiles, which are important factors in preventing coronary heart disease. Certain phospholipids, such as phosphatidylserine, are important for brain health. They are involved in cognitive functions, including memory and learning, and have been studied for their potential to mitigate cognitive decline in aging [5]. Phospholipids also have certain technological characteristics such as emulsifying and foaming capacity [6,7]. Accordingly, different research studies are looking for possibilities to implement buttermilk in traditional productions because of these functional and technological properties [8].

Ultrafiltration (UF) of sweet buttermilk can be useful to improve and accentuate these properties [9,10]. Ultrafiltration is a membrane filtration processing technology that is widely applied in dairy processing for standardization or fractionation purposes and for the development of tailored dairy products [11]. Moreover, UF-concentrated dairy by-products can be directly re-utilized and valorized in dairy plants and contribute significantly to the reuse of materials to minimize waste [12].

Different research studies evaluated the utilization of UF buttermilk in the production of dairy products. The same authors used UF buttermilk for the production of reduced-fat curd cheeses, observing an improvement in textural and sensory characteristics. Poduval and Mistry [13] established that the presence of the fat globule membrane in UF buttermilk, rich in phospholipids, in combination with a concentrated quantity of proteins can reduce the free oil in the production of reduced fat mozzarella cheese because of the improvement in the emulsification capacity. Sweet and cultured, non-ultrafiltered buttermilk has also already been tested to replace milk to produce ice cream [14]. The study highlighted that ice creams made with cultured buttermilk had the highest acidity but also were the most resistant to melting; in general, the authors concluded that the substitution of milk with sweet or cultured buttermilk in ice cream formulation did not cause a deterioration of the product quality and suggested that buttermilk could be considered a suitable raw material for ice cream production [14]. However, to the best of our knowledge, no data can be found on the utilization of UF sweet buttermilk in ice cream production. Given this, the aim of the present work was to investigate the effects of UF sweet buttermilk at different volume reduction ratios (VRR 3 and 5) in the production of a novel clean-label ice cream formulations made with sweet buttermilk-based mixtures.

## 2. Materials and Methods

### 2.1. Materials

Fresh sweet buttermilk with the following composition (fat 1.00 g/100 g, protein 1.90 g/100 g, total solids 6.78 g/100 g), deriving from the production of sweet butter, together with pasteurized cream (fat 39.00 g/100 g, protein 2.30 g/100 g, total solids 43.55 g/100 g) were donated by a local dairy company. Skim milk powder (fat 0.05 g/100 g, protein 35.00 g/100 g, total solids 96.05 g/100 g) was purchased from Radikom Ltd., (Plovdiv, Bulgaria). Saccharose was bought from a local market. The stabilizer/emulsifier used was a commercial ready-to-use mix (Cremodan^®^ SE 334 VEG, Danisco, Copenhagen, Denmark) with the following ingredients: mono- and diglycerides of fatty acids, guar gum, carboxymethylcellulose, and carrageenan.

### 2.2. UF Sweet Buttermilk

The raw sweet buttermilk was pasteurized at 85 °C for 10 min and then it was UF at 50 °C at two different volume reduction ratios (VRR 3 and 5), resulting in a different composition as previously reported in [15]. UF buttermilk with VRR 3 had a fat content of 2.75 g/100 g (determined according to [16]), a protein content of 4.17 g/100 g (determined according to [17]), and a total solids content of 15.18 g/100 g (determined according to [18]); UF buttermilk with VRR 5 had a fat content of 4.60 g/100 g, protein content of 5.69 g/100 g, and a total solids content of 22.64 g/100 g. UF was performed on a laboratory system with a plate and frame module with a filtration area of 1250 cm^2^ [15] and UF25-PAN polyacrylonitrile membrane with a molecular weight cut-off of 25 kDa. The working conditions during the experiments were a transmembrane pressure of 0.2 MPa, a temperature of 50 °C, a feed flow rate of 330 L/h, and a volume reduction ratio of 3 and 5. The concentration factors were defined based on previous findings. The application of a volume reduction ratio (VRR) of 3 showed the best ratio between the permeate flux, the energy demand, and relatively high concentration and rejection factors [15]. On the other hand, the use of a volume reduction ratio of 5 could be valuable to obtain a product with the highest contents of bioactive compounds (proteins, phospholipids, and minerals).

### 2.3. Ice Cream Production

To assess the potential capacity of UF buttermilk in the substitution of additives, four types of ice cream were prepared: S1, ice cream from sweet buttermilk without sweet UF buttermilk and without emulsifier/stabilizer; S2, ice cream from sweet buttermilk with 0.3 g/100 g emulsifier/stabilizer (E/S); S3, ice cream from VRR 3 sweet UF buttermilk without emulsifier/stabilizer; S4, ice cream from VRR 5 sweet UF buttermilk without emulsifier/stabilizer. Samples S1 and S2 were considered as the controls of the study and tested against S3 and S4 to evaluate the effects of the different degrees of UF on the physico-chemical, structural, and sensory properties of the ice cream formulations.

Sweet buttermilk (raw or ultrafiltered), skim milk powder, pasteurized cream, saccharose, and emulsifier/stabilizer mix were mixed in different proportions as reported in Table 1. The ice cream was prepared in accordance with the requirements for high-fat/dessert ice cream formulation [19]. The desired parameters of the obtained mixtures were fat content 10 g/100 g, milk solid nonfat (MSNF) 13 g/100 g, saccharose 14 g/100 g, dry matter 37 g/100 g, and E/S 0.3 g/100 g (only for S2). The ice cream mix was preheated at 60 ± 2 °C, mixed and pasteurized at 70 ± 2 °C for 20 min, and finally cooled at 4 ± 2 °C for 12 h. The mix (0.8 kg for each batch) was then frozen in a discontinuous freezer (Gelato Pro 2000, Nemox International SRL., Pontevico Brescia, Italy) at −12 ± 2 °C for 25 min by continuously mixing with paddles at ~60–70 rpm. The soft ice cream was then filled in cups, hardened in a static freezer at −18 ± 2 °C for 24 h, and kept at the same temperature for storage. The analyses were performed the day after ice cream production.

### 2.4. Determination of Phospholipid Content

A mixture of chloroform and methanol (2:1, *v*/*v*) was used for the extraction of phospholipids from the ice cream. Acetone was used for the precipitation of the phospholipids from the mixture followed by a two-dimensional thin-layer chromatography (TLC) for the isolation of the individual phospholipids [20]. A comparison of the retention factor values with authentic standards was made for the identification. The spots of phospholipids were scraped off and mineralized with sulfuric and perchloric acid, 1:1.

### 2.5. Color Determination of Liquid Ice Cream Mixes

Color measurements were performed using a CR-2600d spectrophotometer (Minolta Co., Osaka, Japan) equipped with a D65 illuminant. An aliquot of sample (10 mL) was transferred in a cylindric glass vial, and colorimetric analyses were performed by placing the spectrophotometer lens in direct contact with the bottom of the vial. Lightness (L * that ranges between 100 of white and 0 of black), redness (a * that ranges between +120 of red and −120 of green), and yellowness (b * that ranges between +120 of yellow and −120 of blue) were measured in SCI mode by considering the CIE L * a * b * color space.

### 2.6. Overrun and Meltdown Rate of Ice Cream

The overrun was determined by comparing the weight of the ice cream mix before and after freezing process. The overrun was calculated in accordance with the following formula suggested in [21]:

(1) $$Overrun\left(\%\right)=\frac{weight\;of\;unfrozen\;mix-weight\;of\;ice\;cream}{weight\;of\;ice\;cream}\cdot 100$$

The meltdown rate was measured by weighting 25 g of ice cream in a plastic strainer with openings of 1 × 1 mm, placed on top of a beaker. The weight of the obtained melted ice cream, collected in the beaker, was measured after 60 min at 20 ± 1 °C. It was measured by applying the following equation [14]:

(2) $$Melt\;down\;rate\left(\%\right)=\frac{weight\;of\;melted\;sample}{weight\;of\;scoop}\cdot 100$$

### 2.7. Ice Cream Texture

For the textural characterization of ice cream samples, a TA.XTplus Texture Analyzer (Stable Micro Systems, Godalming, UK) equipped with a 30 kg load cell and a stainless steel cylindrical probe with a 3 mm diameter was used, according to [22] with slight modifications. Ice cream samples (1 kg) packaged in rectangular plastic boxes were taken out from the freezer (−18 ± 2 °C), and they were immediately subjected to the analysis. A penetration test was performed in quintuplicate, by setting a penetration depth of 20 mm and a penetration rate of 2.0 mm/s. The hardness (N) of the samples was determined as the maximum peak force of the compression cycle, whereas stickiness (N) was referred as the maximum negative peak force during withdrawal [23].

### 2.8. Rheological Analyses

All rheological tests were performed using an MCR102 rheometer (Anton Paar, Gratz, Austria) equipped with a circulating bath (CORIO CD-200F, Julabo Labortechnik GmbH, Seelbach, Germany).

#### 2.8.1. Dynamic Viscosity of Liquid Ice Cream Mixes

The rotational rheological behavior of the liquid ice cream mixes was measured at 4.00 ± 0.01 °C according to the method described in [22] with slight modifications. The rheometer was equipped with a Peltier temperature-controlled cell (mod. C-PTD200, Anton Paar) and a concentric cylinder measuring system (CC27, Anton Paar). Twenty milliliters of sample were poured into the measuring cup, and a hysteresis loop test was performed [24]. The shear rate was initially increased from 10 to 300 s^−1^ in 200 s, then a shear rate of 300 s^−1^ was maintained for 60 s, and finally, the shear rate was decreased from 300 to 10 s^−1^ in 200 s. To explain the ice cream mixes’ rheological behavior, data from the first part of the test (ascending stage) were fitted with the Ostwald–de Waale model (1):

σ = k∙γ^n^ (3)

where k is the consistency coefficient (Pa s^n^) and n is the flow behavior index (dimensionless).

#### 2.8.2. Oscillatory Rheological Behavior of Ice Creams

For this analysis, the rheometer was equipped with a 25 mm crosshatched parallel plate geometry, while temperature control was guaranteed by a Peltier cell (P-PTD 200, Anton Paar). Ice cream disks of 25 mm diameter and ~2 mm height were prepared at −18 °C. After the disk preparation, the sample was kept at −18 ± 2 °C to equilibrate. The parallel plates’ temperature was preset at −15 °C. A sample covered with mineral wool was also used to minimize heat exchange. The ice cream disks were positioned between the parallel plates of the instrument at a fixed gap of 1.5 mm, and the samples were equilibrated with 0 N (± 0.1 N) normal force in the instrument cell for 10 min before analysis. A frequency sweep test was performed at a constant temperature of −15 °C, in the frequency range of 0.1–10 Hz, and at a fixed oscillatory strain of 0.05%. The frequency dependence of the rheological moduli *G*′ and *G*″ was modeled according to [25] by means of power law Equations (4) and (5):

(4) $$G’=k\prime ({f)}^{n^{\prime }}$$

(5) $$G\prime \prime =k\prime \prime ({f)}^{n^{\prime \prime }}$$

where *k*′ and *k*″ coefficients represent the magnitude of *G*′ and *G*″ at a frequency of 1 Hz, while the *n*′ and *n*″ values indicate the dependency of viscoelastic properties on the frequency variation.

Subsequently, an oscillatory thermorheometry (OTR) test was performed according to [26] with slight modifications. The OTR was carried out from −15 to +10 °C by imposing a fixed oscillatory frequency and strain of 10 Hz and 0.05%, respectively. Rheological data were acquired every 30 s imposing a constant temperature ramp rate of 0.5 °C/min.

### 2.9. Ice Cream Sensory Acceptability Test

The sensory acceptability analysis was performed according to [27] by 15 untrained panelists. The considered sensory indexes, evaluated on hedonic scales, were the following: taste—10 points, structure—10 points, consistency—10, color—10 points, and aroma—10 points (maximum overall score—50 points).

### 2.10. Statistical Analysis

The obtained results were treated by one-way ANOVA by using SPSS v.26 (IBM, Armonk, NY, USA). Furthermore, LSD (Least Significant Difference) post hoc analyses were used to perform multiple comparisons. Results were expressed as mean ± SD (standard deviation) of three (n = 3) or four (n = 4) replicates and were rated as significantly different when *p* < 0.05.

## 3. Results and Discussion

### 3.1. Phospholipids Content

The phospholipid content of the four samples is reported in Table 2. The obtained results for total phospholipid content (mg/g of fat) are comparable with the results in [28] where the authors observed 40.4 mg/g of fat for low-phospholipid ice cream mix and 112.3 mg/g of fat for high-phospholipid ice cream mix relative to ice cream with 10% fat content. As expected, the amount of phospholipids increased by increasing the degree of buttermilk concentration. The lowest values were reported for S2, which was probably due to the presence of additives that made it difficult to extract phospholipids for their determination.

The largest share of phospholipids, expressed as % of the total phospholipids, is represented by phosphatidylethanolamine (≈31%), in accordance with previous reports [13]. All other fractions are approximately equal percentages of phosphatidylserine (≈16%), sphingomyelin (≈17%), phosphatidylinositol (≈18%), and phosphatidylcholine (≈17%), respectively. This tendency is similar for all the analyzed samples. The sphingomyelin fraction percentage is also comparable with the data obtained by other researchers for dried phospholipid concentrates, prepared from whey creams [10]. The distribution of phospholipids presented in the milk fat globule membrane is strongly influenced by a number of factors such as the applied technology (i.e., centrifugation, fractionation, regimes for churning the cream) and the seasonality of the raw material. Therefore, the available literature data on the quantitative distribution of phospholipids are very heterogeneous. From the systematic review of Contarini and Povolo [29], it is evident that the relative share of phospholipid species varies widely. For cream, there are reported values of phosphatidylethanolamine (17.7–42.7%), phosphatidylserine (7.2–11.3%), sphingomyelin (20.8–28.6%), phosphatidylinositol (6.8–15.4%), and phosphatidylcholine (14.6–33.7%) [30,31,32], while for buttermilk, they are of the order of phosphatidylethanolamine (8.4–33.5), phosphatidylserine (4.6–10.3%), sphingomyelin (18.3–27.6%), phosphatidylinositol (2.4–8.2%), and phosphatidylcholine (35.5–51.2%) [30,32,33]. It should be considered that both UF buttermilk and cream are involved (in different ratios) in the formulation of ice cream mixes, which explains the obtained results. The values obtained fall within the reported ranges.

### 3.2. Colorimetric and Rheological Characteristics of Liquid Ice Cream Mixes

Color analyses of the non-frozen ice cream mixes revealed significant differences among the different formulations (Table 3). Lightness (L *) and yellowness (b *) showed a decrease following the degree of concentration of buttermilk, as S1 and S2 showed higher L * and b * values compared to S3 and S4 (Table 2). Conversely, greenness (-a *) was found to be higher in the ice cream mixes S3 and S4 containing UF buttermilk compared to S1 and S2. These variations could be caused by the modification of riboflavin content that is naturally present in buttermilk [34,35].

Oscillatory rheological analyses showed a large difference between S4 and the other samples (Table 3). The consistency index (k) of S4 was significantly higher compared to the other ice cream mixes. This result was also in accordance with the hysteresis area, indicative of a thixotropic behavior and a higher degree of organization of the components within the system, for S4 compared to the other samples [24]. Also, the flow behavior index (n) of S4 indicated a stronger pseudoplastic behavior compared to the other three samples that were characterized by a quasi-Newtonian behavior. These results are in agreement with a previous report [36] that observed a predominant pseudoplastic behavior in the case of concentrated buttermilk. Rathnakumar et al. [28], evaluating the effects of phospholipids addition in ice cream mix flow behavior, observed up to 9-fold increase in viscosity and a more pronounced pseudoplastic behavior. This could be possibly related to a greater mix-structuring effect caused by the activity of these components at the air/serum interface, in a similar way to that observed in the case of mono- and di-glycerides [37]. S2 mix was characterized by a slightly but significantly lower n index compared to S1, probably because of the presence of stabilizers and thickeners (guar gum, carrageenan, and carboxy methyl cellulose) that can impact the rheological behavior of the system even at low concentrations [38].

### 3.3. Overrun and Meltdown Rate

The overrun value characterizes the percentage of incorporated air and influences directly ice cream’s functional and sensory properties [39]. Previous reports indicated that sweet buttermilk is characterized by a high emulsifying capacity [7]. In the present study, the overrun value increased with the degree of buttermilk concentration by UF present in the formulation (Table 4). This behavior can be due to the different quantities of phospholipids found in these products and is directly correlated to the higher quantity of incorporated air. Accordingly, the emulsifier/stabilizer usually added in ice cream formulation can be successfully replaced by UF buttermilk. Our obtained data are similar to low and reduced-fat ice creams prepared by Akalın et al. [40], ranging from 25.3% to 39.2%, while Szkolnicka et al. [14] found 55.5% for ice cream made from sweet buttermilk without emulsifiers. At the same time, the obtained results were higher than those reported by Rathnakumar et al. [28] who analyzed ice creams with a similar fat content compared to the present formulations, but with lower (40.41 mg/g fat) or higher (112.29 mg/g fat) phospholipid content (13.48 and 14.11%, respectively). The explanation of these differences can be given by the fact that different approaches are applied in the production of ice cream, as well as the specific formulations of the mixtures. The type of freezer plays probably the most significant role.

The resistance of ice cream to melting is a very important requirement in the production of this dairy product. In our study, a significant difference in the meltdown index among samples was observed. In particular, an inverse relationship between the amount of phospholipids and the degree of ice cream melting was found. Szkolnicka et al. [14] found very similar results for the ice cream sample made from buttermilk without UF concentration (65.9%). El-Kholy et al. [41] established that the substitution of buffalo skim milk for sweet buttermilk caused a significant decrease in the meltdown rate of ice cream.

### 3.4. Textural and Thermo-Rheological Properties of Ice Creams

**Figure 1:**
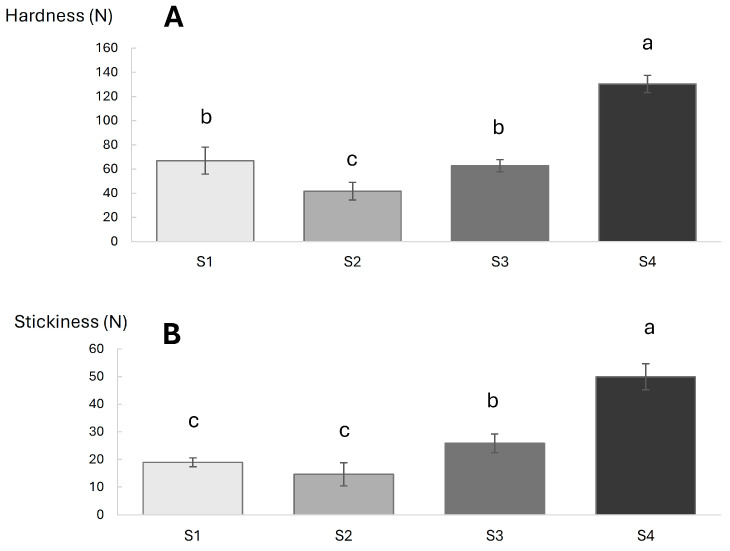
Textural hardness (panel **A**) and stickiness (panel **B**) (expressed in Newton) measured by penetration test (n = 4) of ice cream samples. S1: produced without the addition of emulsifier/stabilizer; S2: produced with the addition of emulsifier/stabilizer; S3: produced with ultrafiltered buttermilk to the degree of concentration 3; S4: produced with ultrafiltered buttermilk to the degree of concentration 5. a–c columns with different superscripts indicate significant differences (*p* < 0.05) according to the post hoc LSD.

Textural properties of ice cream are important parameters that can affect the sensory perception and acceptability of the product, as well as the easiness of use (e.g., scoopability) [42]. Results obtained from the penetration test are reported in Figure 1. As it is possible to observe, the textural hardness of S4 (Figure 1A) was significantly higher compared to the other three samples. S2 showed the lowest hardness values among the evaluated samples, while S1 and S3 reported similar results for this parameter. The lower hardness of S2 compared to S1 could be due to the higher overrun level of S2 (Table 4), leading to a less compact and consistent structure [43]. Conversely, in the case of the samples made with concentrated buttermilk (S3 and S4), the hardness was similar (S3) or significantly higher (S4) to S1, despite the significantly higher overrun values. Also, Prindiville et al. [44] found a negative relation between hardness and overrun percentage. It is important to state that other factors may contribute to defining the textural hardness of ice creams, such as ice crystals’ size, total solids, mix viscosity, and the amount of stabilizers and emulsifiers [28,43]. In accordance with our findings, Rathnakumar et al. [28] observed an increase in hardness in relation to the increase in the phospholipids content of the mix. Also, S4 showed a strongly higher viscosity of the mix compared to the other samples (Table 3) which could impact the different textural hardness of the ice cream [45]. Stickiness (Figure 1B) was significantly different among the samples, leading to a trend that was similar to that observed with hardness; S4 showed the highest stickiness value, followed by S3, while S2 and S1 exhibited the lowest values. Javidi and Razavi [46] also observed a positive correlation between hardness and stickiness.

Power law coefficients (*k′*, *n*′, *n*″, *k*″) of ice cream samples measured at −15 °C by frequency sweeps are reported in Table 5. As it is possible to observe from *k*′ and *k*″ and tan δ values, all the formulations showed a slightly predominant elastic behavior, in accordance with previous reports on ice cream samples [26]. S2 showed significantly higher *k*′, *k*″, and tan δ values compared to the other three samples. Different structural factors are responsible for the definition of the rheological properties of ice creams at freezing temperatures, such as the percentage of overrun, the viscosity of the mix, and the degree of fat destabilization [37,47]. The different rheological behavior of S2 could be explained by the presence of stabilizers that may cause a different structural organization of the ice crystals’ network, as well as the functional role of emulsifiers in the stabilization of the fat phase [26]; those phenomena were also probably great enough to cover the possible negative effect on the rheological properties of the higher overrun of those samples compared to S1. In fact, ice cream with a higher overrun is expected to have a thinner serum phase, which would lead to less contact among ice crystals [47], thus reducing the contribution of the ice crystals’ network on the rheological behavior. In the case of S3 and S4, no difference in terms of rheological parameters was observed at −15 °C in comparison with S1, except for tan δ values between S4 and S1. A different structural organization of the fat phase could be expected in S3 and S4 compared to S1 because of the higher phospholipids content; despite of this, the greater overrun of S3 and S4 may be responsible for covering those rheological differences. S4 showed a lower tan δ value compared to S2 and S3, indicating a higher elastic-to-viscous behavior ratio of the sample that is coherent with the greater measured textural hardness (Figure 1).

**Figure 2:**
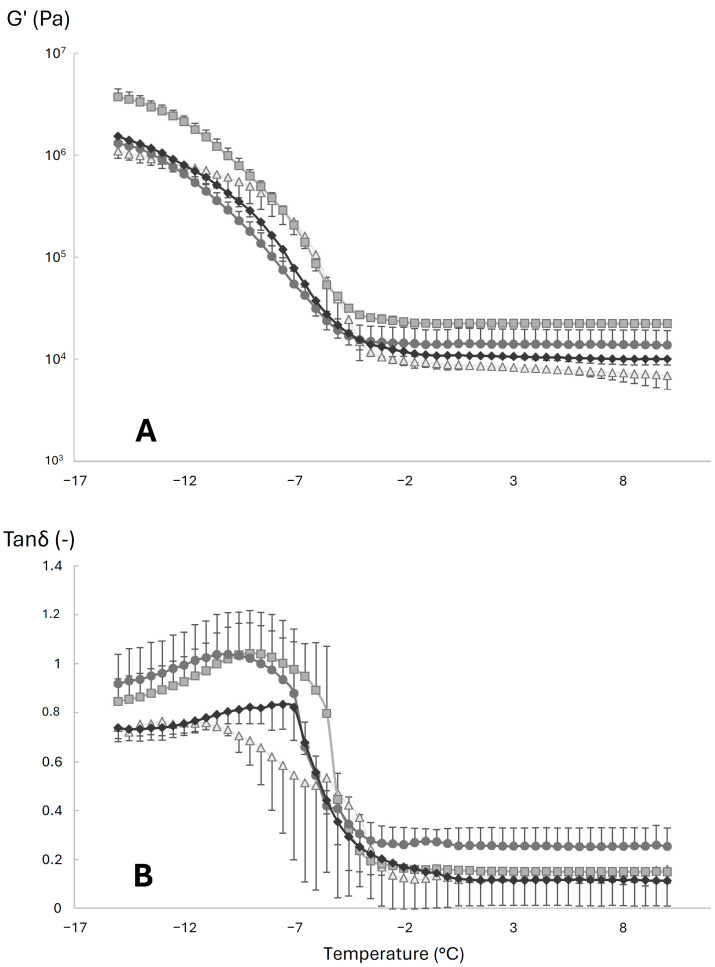
Evolution of storage modulus (*G*′) (panel **A**) and tan δ (panel **B**) of ice cream samples subjected to the temperature ramp test (from −15 °C to 10 °C, strain 0.05%, frequency 10 Hz) (n = 3). S1 (triangles): ice cream produced with sweet buttermilk, without the addition of emulsifier/stabilizer; S2 (squares): produced with sweet buttermilk, with the addition of emulsifier/stabilizer; S3 (circles): produced with sweet buttermilk UF to 3 degrees of concentration; S4 (diamonds): produced with ultrafiltered buttermilk to 5 degrees of concentration.

The thermo-rheological behavior of ice cream samples during melting is depicted in Figure 2. In accordance with previous literature data [26,28,48], the curve can be divided into three zones: zone I (−15 °C < T < −10 °C) where the rheological behavior is dominated by the ice crystals’ network, zone II (−10 °C < T < 0 °C) corresponding to the ice melting temperature range, and zone III (0 °C < T < 10 °C) showing the rheological behavior of the remnant foam that remains after ice melting. In zone I, the differences among the samples were similar to those detected with frequency sweeps tests performed at −15 °C (Table 5), as in this temperature range, the behavior is still governed by the arrangement of ice crystals within the matrix and the ice melting phenomena are still limited. In the temperature range between the transitions from zone I to zone II, it is possible to observe a slight decrease in the storage modulus (Figure 2A) and an increase in tan δ resulting in a peak (Figure 2B). In particular, in the case of S2 and S3, the increase in tan δ was greater than S4 and S1, respectively, causing a cross-over between *G*′ and *G*″ (tan δ > 1); also, the peak of tanδ in S2 and S3 was shifted at lower temperatures compared to the other two samples. Tsevdou et al. [49] attributed the tan δ peak (measured in a temperature range around −14 and −17 °C, with shouldered peaks around −8/−5 °C) to an increase in the molecular mobility of unfrozen water in the freeze-concentrated serum phase. However, in our experiment, since the initial temperature was higher, the observed peak in tan δ may be related to both changes in the molecular mobility of the serum phase and to the ice melting phenomena [26,48]. Liu et al. [47] observed a negative shift of the initial melting temperature of ice cream formulations with higher overrun, because of the loose connectivity of ice crystals within the matrix. In zone II, ice cream samples showed a marked decrease in rheological moduli that was related to the loss of the ice crystals’ structure. The decrease in rheological moduli (as observable for *G*′, Figure 2A) was more pronounced for S1 and S2 compared to the samples containing the UF buttermilk. These results are also in agreement with the meltdown index (Table 4). This different melting behavior can be related to the structural role of the lipid network [47], which can be modified and enhanced by a higher content of phospholipids in the mix. Accordingly, also Rathnakumar et al. [28] observed a delayed meltdown in the ice cream formulated with a high phospholipids content (~112 mg/g of fat). Also the lower overrun, especially for S1, can cause this different behavior, since a higher overrun mainly slows down melting while a lower overrun contributes to a faster melting rate [47]. This different melting behavior was also quite evident from the steep decrease in tan δ within the temperature range of zone II; conversely, S1 showed a lower rate of decrease in this parameter, probably because of the lower values of overrun. In zone III, after the end of the ice-melting process, the network governed by the fat aggregates and hydrocolloids within the serum phase becomes rheologically dominant. Accordingly, in all the samples, a plateau for *G*′ and tan δ was observed. The storage modulus of S1 in this plateau region was significantly lower (*G*′ at 0 °C ~9 kPa) compared to the other three samples (*G*′ at 0 °C~20 kPa for S2). This can be explained by a lower content of phospholipids (compared to S3 and S4) and the absence of thickeners and stabilizers (compared to S2) [26].

### 3.5. Sensory Acceptability Test

**Figure 3:**
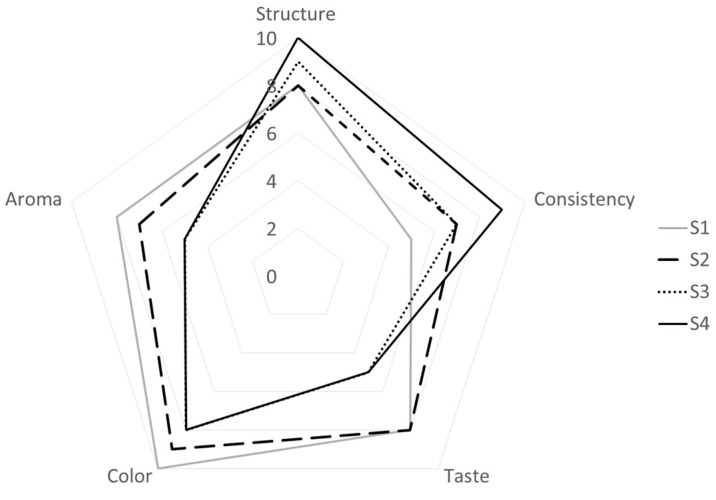
Results for the sensory analysis of different ice cream samples. S1 (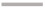): ice cream produced with sweet buttermilk, without the addition of emulsifier/stabilizer; S2 (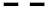): produced with sweet buttermilk, with the addition of emulsifier/stabilizer; S3 (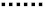): produced with sweet buttermilk UF to 3 degrees of concentration; S4 (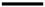): produced with ultrafiltered buttermilk to 5 degrees of concentration. In the note scale the points are awarded from 0 to 10, where 0 (zero) corresponds to unsatisfactory and 10 (ten) to very good.

The acceptability results are reported in Figure 3. Formulation S4 and S3 were characterized by the most appreciated structure; S4 was also the most appreciated sample in terms of consistency. This can be explained by the fact that these samples are characterized by a higher phospholipids content (Table 2), which are known to improve the structure and consistency of the product, but according to Goff and Hartel [50], this results in a drier ice cream with smoother body and texture. The taste and aroma of S4 and S3 were significantly less appreciated than the other samples. The color of the S4 sample was significantly different from the other three samples, in accordance with the instrumental color measurements (Table 3), and was less appreciated by the consumers. Also, S4 and S3 possessed a less appreciated taste and aroma compared to S1 and S2 where buttermilk was not concentrated by UF. This can be due to some enzymatic hydrolysis from residual psychrophilic or psychotropic microorganisms during the ripening of the ice cream mix as suggested in [51], or some peptides that are originally present in buttermilk that may cause off-tastes and off-flavors (i.e., oxidized, bitter). This tendency was already observed in the case of Cheddar cheese made with UF buttermilk [52] and Pizza cheese made with sweet buttermilk [53]. Zhao et al. [54] prepared a low-fat yogurt with buttermilk and observed that the addition of buttermilk improved the sensory profile of the product, but an excessive addition (>4%) decreased its acceptability with respect to flavor. Szkolnicka et al. [14] found that sweet buttermilk ice cream was characterized by sensory properties similar to the control sample, prepared from milk. El-Kholy et al. [41] found that ice cream made from sweet buttermilk had a better flavor, texture, appearance, and color compared to ice cream made from buffalo skim milk. Our results confirm that buttermilk-based ice cream is characterized by acceptable sensory properties.

## 4. Conclusions

The results obtained in this study showed that the utilization of ultrafiltered (UF) sweet buttermilk in ice cream production can influence different techno-functional and rheological properties of the product. The increase in the degree of concentration caused an increase in the overrun and a decrease in the meltdown rate, as well as a modification in the melting profile. Also, the utilization of UF sweet buttermilk promoted changes in the structural and textural characteristics of the ice creams, improving the body and consistency of ice cream as observed both with instrumental tests and sensory analyses. On the other hand, the utilization of UF buttermilk had a negative effect on taste, aroma, and color. This study demonstrated that UF can be a valuable technology for the valorization of buttermilk in novel ice cream products made with sweet buttermilk-based mixtures, with the aim to replace the structural role of emulsifiers and stabilizers, but attention should be paid to the negative effect on some sensory characteristics.

## Figures and Tables

**Table 1:** Amount of ingredients (expressed as g/100 g) used in the ice cream mix preparation.

Ingredient (g/100 g Ice Cream Mix)	S1	S2	S3	S4
Sweet buttermilk	52.1	51.9	-	-
Dry skim milk	9.8	9.8	5.0	-
Fresh cream	24.0	24.0	20.0	17.0
Saccharose	14.0	14.0	14.0	14.0
UF sweet buttermilk (VRR 3)	-	-	61.0	-
UF sweet buttermilk (VRR 5)	-	-	-	69.0
Emulsifier/Stabilizer mix	-	0.3	-	-

-: not added; VRR: volume reduction ratio. S1: ice cream produced with sweet buttermilk, without the addition of emulsifier/stabilizer; S2: produced with sweet buttermilk, with the addition of emulsifier/stabilizer; S3: produced with sweet buttermilk UF to 3 degrees of concentration; S4: produced with ultrafiltered buttermilk to 5 degrees of concentration.

**Table 2:** Phospholipid content of ice cream samples.

Phospholipids, mg/g Product	S1	S2	S3	S4
Phosphatidylserine (PS)	1.01 ± 0.04 ^a^	0.86 ± 0.02 ^b^	1.22 ± 0.00 ^c^	1.57 ± 0.01 ^d^
Phosphatidylinositol (PI)	1.19 ± 0.03 ^a^	0.95 ± 0.05 ^b^	1.33 ± 0.06 ^c^	1.75 ± 0.09 ^d^
Phosphatidylcholine (PC)	1.09 ± 0.07 ^a^	0.84 ± 0.07 ^b^	1.29 ± 0.05 ^c^	1.61 ± 0.05 ^d^
Phosphatidylethanolamine (PE)	2.03 ± 0.06 ^a^	1.82 ± 0.01 ^b^	2.25 ± 0.12 ^c^	2.57 ± 0.13 ^d^
Sphingomyelin (SM)	1.12 ± 0.04 ^a^	0.86 ± 0.04 ^b^	1.14 ± 0.08 ^c^	1.71 ± 0.02 ^d^
Total phospholipids, mg/g product	6.43 ± 0.21 ^a^	5.10 ± 0.09 ^b^	7.41 ± 0.07 ^c^	9.77 ± 0.25 ^d^
Total phospholipids, mg/g fat	79.95 ± 2.62 ^a^	66.72 ± 1.15 ^b^	96.89 ± 0.94 ^c^	113.9 ± 2.95 ^d^

a–d Means ± SD (n = 4) within columns with different superscripts indicate significant differences (*p* < 0.05) according to the LSD method. S1: ice cream produced with sweet buttermilk, without the addition of emulsifier/stabilizer; S2: produced with sweet buttermilk, with the addition of emulsifier/stabilizer; S3: produced with sweet buttermilk UF to 3 degrees of concentration; S4: produced with ultrafiltered buttermilk to 5 degrees of concentration.

**Table 3:** Results of colorimetric and rotational rheological analyses performed on ice cream mixed sample at 25 °C.

Sample	L *	a *	b *	k (Pa s)	n (-)	Hysteresis Area (Pa)
S1	85.59 ± 0.02 ^a^	−2.82 ± 0.04 ^a^	7.09 ± 0.03 ^b^	0.02 ± <0.01 ^b^	0.966 ± 0.030 ^a^	0.21 ± <0.01 ^b^
S2	85.02 ± 0.03 ^b^	−2.87 ± 0.01 ^a^	7.61 ± 0.04 ^a^	0.26 ± 0.01 ^b^	0.833 ± 0.022 ^b^	1.79 ± 0.20 ^b^
S3	83.68 ± 0.01 ^c^	−3.69 ± 0.02 ^c^	5.60 ± 0.02 ^c^	0.07 ± 0.01 ^b^	0.915 ± 0.016 ^ab^	0.61 ± 0.05 ^b^
S4	82.52 ± 0.07 ^d^	−3.40 ± 0.03 ^b^	4.43 ± 0.09 ^d^	26.22 ± 5.42 ^a^	0.286 ± 0.031 ^c^	50.83 ± 19.30 ^a^

a–d Means (n = 3) within columns with different superscripts indicate significant differences (*p* < 0.05); L *—lightness; a *—redness; b *—yellowness; k—consistency coefficient; n—flow behavior index. S1: ice cream produced with sweet buttermilk, without the addition of emulsifier/stabilizer; S2: produced with sweet buttermilk, with the addition of emulsifier/stabilizer; S3: produced with sweet buttermilk UF to 3 degrees of concentration; S4: produced with ultrafiltered buttermilk to 5 degrees of concentration.

**Table 4:** Results for overrun and meltdown analyses of different ice cream samples.

Sample	S1	S2	S3	S4
Overrun, %	23.7 ± 1.7 ^d^	30.8 ± 0.8 ^c^	33.3 ± <0.1 ^b^	35.8 ± 0.8 ^a^
Melting, %	77.2 ± 1.1 ^d^	66.2 ± 0.9 ^c^	49.7 ± 1.3 ^b^	43.4 ± 0.9 ^a^

a–d Means ± SD (n = 4) within columns with different superscripts indicate significant differences (*p* < 0.05) according to the LSD method. S1: ice cream produced with sweet buttermilk, without the addition of emulsifier/stabilizer; S2: produced with sweet buttermilk, with the addition of emulsifier/stabilizer; S3: produced with sweet buttermilk UF to 3 degrees of concentration; S4: produced with ultrafiltered buttermilk to 5 degrees of concentration.

**Table 5:** Power law oscillatory rheological parameters (*k*′, *n*′, *k*″, *n*″, and tan δ) obtained from frequency sweep analyses of ice cream samples at −15 °C.

Sample	*k*′ (kPa)	*n*′ (-)	*k*″ (kPa)	*n*″ (-)	tan δ (1 Hz)
S1	677 ± 240 ^b^	0.3073 ± 0.0161 ^a^	586 ± 197 ^b^	0.2296 ± 0.0547 ^a^	0.8689 ± 0.0121 ^bc^
S2	1977 ± 372 ^a^	0.3077 ± 0.0249 ^a^	1919 ± 296 ^a^	0.2236 ± 0.0015 ^a^	0.9735 ± 0.0235 ^a^
S3	722 ± 126 ^b^	0.2775 ± 0.0784 ^a^	674 ± 156 ^b^	0.2580 ± 0.0558 ^a^	0.9289 ± 0.0376 ^ab^
S4	905 ± 12 ^b^	0.3110 ± 0.0002 ^a^	746 ± 2 ^b^	0.2369 ± 0.0398 ^a^	0.8245 ± 0.0089 ^c^

a–c Means (n = 3) within columns with different superscripts indicate significant differences (*p* < 0.05). S1: ice cream produced with sweet buttermilk, without the addition of emulsifier/stabilizer; S2: produced with sweet buttermilk, with the addition of emulsifier/stabilizer; S3: produced with sweet buttermilk UF to 3 degrees of concentration; S4: produced with ultrafiltered buttermilk to 5 degrees of concentration.

## Data Availability

The original contributions presented in the study are included in the article, further inquiries can be directed to the corresponding author.
